# A descriptive study on evaluation of bio-medical waste management in a tertiary care public hospital of North India

**DOI:** 10.1186/2052-336X-12-69

**Published:** 2014-04-17

**Authors:** Rajiv Kumar, Anil Kumar Gupta, Arun Kumar Aggarwal, Ashok Kumar

**Affiliations:** 1 Department of Hospital Administration, Post-Graduate Institute of Medical Education and Research, Chandigarh, India; 2 School of Public Health, Post-Graduate Institute of Medical Education and Research, Chandigarh, India

**Keywords:** Biomedical waste, Medical waste, Environment, Waste receptacles, Segregation of waste, Mutilation of recyclable waste, Disinfection of waste

## Abstract

**Background:**

Proper management of Biomedical waste (BMW) generated in a healthcare facility is one of the most important functions of a healthcare worker (HCW) as its improper management not only poses risk to human beings and environment, but may also invite legal action against HCW as well as hospital administration. This study was carried out to evaluate quality of BMW management in 1100-bedded hospital attached to a tertiary care public institute in North India.

**Methods:**

A checklist, including 29 parameters related to various functions to be carried out at source of generation by a HCW for BMW management was prepared by researcher and used after validation to record observations in all the 70 areas of hospital. A total of 6 visits were made to each area and mean percentage score was calculated for each area and each category of waste management.

**Results:**

It was found that summated mean percentage score of ‘Treatment Room of Wards’, which were used exclusively by resident doctors, was significantly lower than Operation Theatres (p value: 0.033) and ‘Central Waste Collection Points of Wards’ (p value: 0.018) for the function of ‘mutilation of recyclable waste’ and it was significantly lower than all other areas (p value: 0.006 to 0.017) for the function of ‘disinfection of waste’.

**Conclusion:**

It is concluded that more emphasis needs to be laid on ‘mutilation of recyclable waste’ and disinfection of waste’ by HCWs especially resident doctors.

## Introduction

When patient care activities are carried out in a healthcare setting, certain waste is produced which has the potential to cause harm to human beings and environment. Such waste includes soiled cotton, bandages, hypodermic needles, syringes, tubings such as intravenous sets, and urinary catheters etc. Such waste is commonly called as bio-medical waste (BMW) in India, though it is also known by various other names such as clinical waste, medical waste and health-care waste in different parts of world. Such waste constitutes merely 15 to 25% of total waste generated in a hospital, the remaining being general waste such as waste paper, wrapper of drugs, cardboard and left-over food etc [1]. The general waste is treated by local municipality in same way as house-hold waste, but special precautions and treatment modalities are required for BMW, so that it does not cause any harm to human beings and environment [2]. Though as many as 40 pathogens have been documented to be transmitted by BMW [3], its well documented propensity to cause transmission of 3 pathogens namely Human Immunodeficiency Virus (HIV), Hepatitis B Virus (HBV) and Hepatitis C Virus (HCV) makes it essential that due care is exercised while handling and disposing it [1].

The enacted legislations in various countries have made it mandatory for a healthcare facility to manage its waste properly. In India, the legislation governing BMW management is called as Bio-Medical Waste (Management and Handling) Rules, 1998 [4] and has been promulgated under Environment (Protection) Act, 1986 [5].

Due to propensity of BMW to transmit pathogens mentioned above and the risk of inviting adverse legal action, it is one of the top most priorities for a Hospital Administrator to put in place a framework for its proper management as well as to keep a close watch over the waste management practices being followed by Health Care Workers (HCWs) and waste handlers.

There are primarily 4 broad functions for BMW management at source of generation, viz. placement of waste receptacles or bins lined with waste bags at source of generation, segregation of waste, mutilation of recyclable waste and disinfection of waste [1,2,4].

It is highly desirable for a Hospital Administrator to know the weak points in the chain of waste management so that these could be addressed appropriately.

Keeping this in view, the present study was conducted with the aim to evaluate BMW management practices at source of generation in an1100-bedded hospital of a tertiary care referral public hospital of North India.

## Methods

### Ethical clearance

Ethical clearance from institute’s ethics committee and permission from Medical Superintendent to collect data from various patient care areas was taken before the study. The study was approved by 'Thesis Review Committee’ of Post-Graduate Institute of Medical Education and Research (PGIMER), Chandīgarh, India.

### Study areas and number of visits

The study was conducted between December 2009 and April 2010. No sampling was done and all 70 patient care areas of 1100-bedded hospital were included in study. These areas were: Emergency Areas (12 in number), Waste Collection Points of Wards (25 in number), Treatment Rooms of Wards (11 in number), Intensive Care Units (8 in number) and Operation Theatres (14 in number).

Each area was visited on any 3 non-consecutive days in the study period. No visit was made on Sundays and on Public Holidays. Areas were visited during morning hours between 7 am to 10 am and evening hours of the same day between 2 pm and 4 pm. Thus a total of 6 visits were made to each area. The chosen timings were such when maximum BMW is generated in a patient care area as this was the time when blood samples of patients were taken and medication injections were given. Although medication injections were also given during evening hours and night hours, such time period was excluded from the study due to operational difficulties in collecting data during these timings. All observations were made by same researcher.

### Study tool

Data were recorded on a researcher made checklist covering various aspects of BMW management at source of generation of waste.

Primarily, 4 broad functions are carried out at source viz. (i) placement of 4 colour-coded i.e. black, yellow, red and blue waste bins which are lined on inner side by similarly coloured waste bags; (ii) segregation of waste in such waste bags i.e. general waste like waste paper, wrapper of drugs, cardboard, left-over food etc. is to be put into black; soiled infected waste like dressing material, cotton swabs etc. is to be put into yellow; plastic waste like plastic syringes, dextrose bottles, intravenous sets, Ryle’s tubes, urinary catheters etc. is to be put into red and sharps like hypodermic needles, surgical blades, glass etc. is to be put into blue bags (iii) mutilation of recyclable waste like disposable syringes, plastic dextrose bottles, plastic tubings and hypodermic needles and (iv) disinfection of certain categories of waste notably plastics and sharps [1,2,4]. In the hospital, electrically operated needle cutters were used to mutilate hypodermic needles and nozzle (hub) of disposable syringes and scissors were used to cut the plastic tubings and 1% bleaching powder was used to disinfect plastics and sharps. Parameters related to each of the 4 main categories mentioned above were identified and a checklist was prepared (Table 1). Each desirable observation was assigned ‘1’ mark and each undesirable observation was assigned ‘0’ mark. There were some parameters, observations regarding which could be in part desirable and in part undesirable in a given area, such observation was assigned ‘0.5’ mark. As an example, if all of the used hypodermic needles in an area were found mutilated (desirable), it was assigned ‘1’ mark; if none of the needles was mutilated (undesirable), it was assigned ‘0’ mark and if some of the needles were mutilated and some not, such observation was assigned ‘0.5’ mark. The checklist was tested in another patient care area of institute not included in the study namely Advanced Pediatrics Centre and parameters which were not feasible to observe were deleted from checklist. In the final score-sheet, there were 16 parameters noted under category ‘condition of waste receptacles’, 4 parameters noted under category ‘segregation of waste’, 6 parameters noted under category ‘mutilation of recyclable waste’ and 3 parameters noted under category ‘disinfection of waste’. Thus a total of 29 parameters were noted in each study unit.

**Table 1:** Sample checklist for bio-medical waste management practices in patient care areas

***Name of ward/area***				***Date and time of observation***			
**S. no.**	**Parameters**	**Observation**		**S. no.**	**Parameters**	**Observation**	
		**Yes**	**No**			**Yes**	**No**
**A**	**Condition of waste receptacles**						
1	Is black colored waste bin available in ward?			2	Is yellow colored waste bin available in ward?		
3	Is red colored waste bin available in ward?			4	Is blue colored waste bin available in ward?		
5	Has black bag been placed lining the inner side of black bin?			6	Has yellow bag been placed lining the inner side of yellow bin?		
7	Has red bag been placed lining the inner side of red bin?			8	Has blue bag been placed lining the inner side of blue bin?		
9	Is black bag securely fitted with the bin?			10	Is yellow bag securely fitted with the bin?		
11	Is red bag securely fitted with the bin?			12	Is blue bag securely fitted with the bin?		
13	Are waste bins covered?			14	If covered, is cover foot-operated?		
15	Is the biohazard symbol imprinted over waste bags?			16	Are posters to guide users displayed near waste bins?		
**B**	**Segregation of waste**						
17	Does black bag contain only general waste?			18	Does yellow bag contain only soiled infected waste?		
19	Does red bag contain only plastic waste?			20	Does blue bag contain only sharps waste?		
**C**	**Mutilation of recyclable waste**						
21	Are used hypodermic needles destroyed?			22	Is nozzle of used syringes destroyed?		
23	Are used hypodermic needles found re-capped?			24	Are used hypodermic needles found bent?		
25	Are used plastic bottles cut?			26	Are used plastic tubings cut?		
**D**	**Disinfection of plastics and sharps**						
27	Is disinfectant solution put into red containers?			28	Is disinfectant solution put into blue containers		
29	Is barrel and plunger of syringe separate before immersion into disinfectant solution?						

### Data analysis

The score obtained in 6 visits for a particular category of waste management was divided by 6 to obtain the mean score and then percentage mean score was calculated. The score of all observation units in a given area was summated and mean percentage score of the area was calculated. This was done for all categories of waste management and for all areas.

The statistical analysis was carried out using Statistical Package for Social Sciences (SPSS Inc., Chicago, IL, version 15.0 for Windows). All quantitative variables were estimated using measures of central location (mean, median) and measures of dispersion (standard deviation, standard error and 95% confidence interval). Means were compared using One-way ANOVA (analysis of variance) where there were more than two groups and unpaired *t*-test where there were two groups. P-value ≤ 0.05 was used as a cut point to determine significance.

## Results

The overall mean percentage score for BMW management at source of generation of waste was 88%. Category-wise, the mean percentage score of ‘condition of receptacles’ was 87%, ‘waste segregation’ was 96%, ‘mutilation of recyclable waste’ was 88% and ‘disinfection of waste’ was 81%.

Area-wise, the mean percentage score of Emergency was 88%, Central Waste Collection Points of Wards’ was 89%, ‘Treatment Room of Wards’ was 81%, OTs was 90% and ICUs was 92% (Table 2).

**Table 2:** Mean percentage score of different categories of BMW management in different areas at source of generation of waste

**Category of BMW management**	**Emergency**	**Central waste collection area of wards**	**Treatment room of wards**	**Operation theatres**	**Intensive care units**	**Overall score of category of BMW management**
**Condition of waste receptacles**	**87%**	**87%**	**85%**	**87%**	**88%**	**87%**
	(95% CI: 86 to 88%)	(95% CI: 86 to 88%)	(95% CI: 81 to 88%),	(95% CI: 87 to 88%)	(95% CI: 86 to 90%)	(95% CI: 86 to 87%)
**Waste segregation**	**92%**	**96%**	**96%**	**96%**	**100%**	**96%**
	(95% CI: 88 to 97%)	(95% CI: 92 to 99%)	(95% CI: 92 to 100%)	(95% CI: 92 to 100%)	(95% CI: 100 to 100%)	(95% CI: 94 to 98%)
**Mutilation of recyclable waste**	**85%**	**90%**	**80%**	**92%**	**92%**	**88%**
	(95% CI: 82 to 88%)	(95% CI: 85 to 95%)	(95% CI: 74 to 86%)	(95% CI: 86 to 98%)	(95% CI: 85% to 99%)	(95% CI: 86 to 91%)
**Disinfection of waste**	**86%**	**82%**	**63%**	**85%**	**88%**	**81%**
	(95% CI: 80 to 92%)	(95% CI: 75 to 90%)	(95% CI: 52 to 74%)	(95% CI: 75 to 94%)	(95% CI: 77 to 75%)	(95% CI: 77 to 85%)
**Overall score of area**	**88%**	**89%**	**81%**	**90%**	**92%**	**88%**
	(95% CI: 85 to 90%)	(95% CI: 85 to 92%)	(95% CI: 76 to 86%)	(95% CI: 86 to 95%)	(95% CI: 88 to 96%)	(95% CI: 86.20 to 89.76%).

### Score of each category in each area

In emergency, the mean score for ‘condition of waste receptacles’, ‘segregation of waste’, ‘mutilation of recyclable waste’ and ‘disinfection of waste’ was 87%, 92%, 85% and 86% respectively. For Central Waste Collection Points of Wards, the score for these categories was 87%, 96%, 90% and 82% respectively; for Treatment Room of wards the score was 85%, 96%, 80% and 63% respectively; for OTs, the score was 87%, 96%, 92% and 85% respectively and for ICUs, the score was 88%, 100%, 92% and 88% respectively.

### Comparison of scores amongst different areas

The comparison of scores of different areas showed that score related to ‘condition of waste receptacles’ and ‘segregation of waste’ was not significantly different amongst various areas i.e. Emergency areas, Central Waste Collection Points of Wards, Treatment Room of Wards, OTs and ICUs.

However, the score regarding ‘mutilation of recyclable waste’ and ‘disinfection of waste’ was significantly different amongst various areas (Table 3).

**Table 3:** Level of significance of difference in scores of different categories of BMW management amongst different areas

**S. no.**	**Category**	**Level of significance of difference in scores amongst different areas using one-way ANOVA**
1	Condition of waste receptacles	Not significant
		(p value: 0.077)
2	Segregation of waste	Not significant
		(p Value: 0.230)
3	Mutilation of recyclable waste	Significant
		(p value: 0.016)
4	Disinfection of waste	Highly Significant
		(p value: 0.004)

The score regarding ‘mutilation of recyclable waste’ was found significantly different between OTs and Treatment Room of wards. The score in OTs (n: 13, mean: 92%, 95% CI: 86 to 98%) was significantly higher (p value: 0.033) than that in Treatment Room of Wards (n: 11, mean: 80%, 95% CI: 74 to 86%).

The score related to ‘disinfection of waste’ in Treatment Room of Wards (n: 11, mean: 63%, 95% CI: 52 to 75%) was significantly lower than all other areas. It was significantly lower than Emergency (n: 11, mean: 86%, 95% CI: 80 to 92%, p value: 0.016); Central Waste Collection Points of Wards (n: 25, mean: 82%, 95% CI: 75 to 90%, p value: 0.016); OTs (n: 13, mean: 85%, 95% CI: 75 to 94%, p value: 0.017) and ICUs (n: 8, mean: 88%, 95% CI: 77 to 98%, p value: 0.017).

The score of Central Waste Collection Points of Ward (n: 25, mean: 90%, 95% CI: 85 to 95%) was significantly higher (p value: 0.018) than that in Treatment Room of Ward (n: 11, mean: 80%, 95% CI: 74 to 86%) with respect to ‘mutilation of recyclable waste’. The score regarding ‘disinfection of waste’ was also significantly higher (p value: 0.006) in Central Waste Collection Points of Wards (n: 25, mean: 82%, 95% CI: 75 to 90%) as compared to that in Treatment Room of Wards (n: 11, mean: 63%, 95% CI: 52 to 75%) (Table 4).

**Table 4:** Level of significance of lower score in Treatment Room of Wards v/s other areas regarding ‘mutilation of recyclable waste’ and ‘disinfection of waste’

**S. no.**	**Category**	**Area**	**Level of significance using unpaired *t*-test**
1	Mutilation of recyclable waste	Treatment Room of wards v/s OTs	p value: 0.033
		Treatment Room of wards v/s Central Waste Collection Points of Wards	p value: 0.018
2	Disinfection of waste	Treatment Room of wards v/s Emergency	p value: 0.016
		Treatment Room of wards v/s Central Waste Collection Points of Wards	p value: 0.006
		Treatment Room of wards v/s OTs	p value: 0.017
		Treatment Room of wards v/s ICUs	p value: 0.016

### Score of individual parameters

The summated mean percentage score of each of 29 observed parameters showed that it was 100% for placement of waste receptacles and 0% for ‘is cover on waste receptacle foot-operated’; for segregation of waste in various waste receptacles, it was from 84.84% to 98.93%; for destruction of used needles and nozzle of syringes, it was 91.21% and 85.73% respectively and for putting of disinfectant solution in blue and red bags, it was 78.97% and 78.68% respectively (Table 5).

**Table 5:** Score of individual parameters that constituted four broad categories of BMW management at source in all study areas in descending order

**S. no.**	**Parameter**	*N	**% age mean score**	**S. no.**	**Parameter**	*N	**% age mean score**
1	Placement of black bins	420	100	2	Placement of yellow bins	420	100
3	Placement of red bins	420	100	4	Placement of blue bins	420	100
5	Imprinting of biohazard symbol over waste bags	420	100	6	Bending of needles manually	408	100
7	Placement of red bags	420	99.81	8	Are red bags securely fitted	420	99.81
9	Displaying of posters to guide users	408	99.42	10	Segregation of waste in blue bags	420	98.93
11	Segregation of waste in black bags	420	98.71	12	Placement of blue bags	420	97.79
13	Are blue bags securely fitted	420	97.79	14	Placement of black bags	420	96.91
15	Are black bags securely fitted	420	96.91	16	Are used needles re-capped	408	94.89
17	Segregation of waste in red bags	420	94.63	18	Placement of yellow bags	420	91.63
19	Are yellow bags securely fitted	420	91.63	20	Are used needles destroyed	408	91.21
21	Are nozzles of used syringes destroyed	408	85.73	22	Segregation of waste in yellow bags	420	84.84
23	Putting of disinfectant solution in blue bags	408	78.97	24	Putting of disinfectant solution in red bags	408	78.68
25	Cutting of used plastic bottles	408	73.60	26	Separation of barrel and plunger of syringes	408	71.90
27	Cutting of used tubings	408	68.33	28	Are waste bins covered	420	2.28
29	If covered, are covers foot operated	420	0.00				

*N = number of observations.

## Discussion

Segregation of waste is the most crucial step for proper management of BMW as waste segregated into various colour-coded containers is finally taken to different sites for disposal. Presence of a wrong kind of waste in a particular container will obviously nullify the efforts of appropriate disposal of waste. This implies that for proper segregation of waste, the waste bins in appropriate number, at appropriate places and with appropriate colour-code are required to be placed at the source of generation of waste.

The summated score of ‘condition of waste receptacles’ in all the patient care areas was more than 80%. Various studies have found poor condition of waste receptacles for waste disposal. In a 600-bedded super-specialty corporate hospital of a South Indian city, there were only white receptacles for all types of BMW for aesthetic reasons and since the colour of all receptacles or bins was same, following the segregation practices was difficult [6]. In studies in Irbid city of Jordan [7] and UK [8], waste bins or receptacles were found to be in poor shape.

The high score of ‘condition of waste receptacles’ in all patient care areas in present study implies that the basic infrastructure for proper segregation of waste at the source of generation of waste was well placed in hospital. However, it was found that almost all waste receptacles were open i.e. without any lid over them. Waste receptacles should preferably be covered ones having foot-operated lids [1] and so it is desirable to gradually replace the existing open type waste receptacles with the ones having foot-operated lids.

High score for ‘segregation of waste’ (96%) shows that this crucial aspect of waste management was being appropriately addressed. In a study in 1800-bedded tertiary care hospital in Mumbai [9], it was found that waste segregation was less than satisfactory in 40.3% of areas in spite of continuous monitoring and informal counselling of HCWs. In another study conducted in 1300-bedded Government College and Hospital and 50-bedded private hospital of a south Indian city [6], it was found that waste segregation was not proper. However, the number of areas where it was not proper has not been mentioned in the study. In a study in Jordan also [7], it was found that waste segregation practices were non-existent in spite of existence of a regulatory framework. In studies conducted in Egypt [10], England [8] and Ethiopia [11] also, the waste segregation practices were found to be poor. In a study in a 350-bedded polyclinic at Lucknow, India [12] and 574-bedded tertiary care Medical Institute located at Belgaum, Karnataka, India [13], the waste segregation practices were found to be good. However, the authors did not mention the exact percentage of areas where segregation practices were found good.

As segregation of BMW is the most crucial aspect of BMW management, still more focus may need to be laid in certain areas of hospital for this aspect of waste management particularly in Emergency areas as there the cumulative score (92%) was relatively less as compared to other areas of hospital, though this difference was not statistically significant. High score in ICUs may be due to relatively good staff to patient ratio whereas over-crowding of patients in Emergency and relatively less favourable staff to patient ratio may be the cause for relatively lower score in Emergency areas. As hospital does not have autoclave and instead has only 02 incinerators for terminal disposal of waste and incineration is considered a relatively poor technology from point of view of environmental safety and long term cost [14], it makes it essential that still more focus is laid on proper segregation of waste so that waste not meant for incineration like plastic material etc. could be prevented to mix into waste stream meant for incineration.

In the hospital under study, electrically operated needle cutters are used to mutilate the used needles and nozzle (hub) of used syringes and scissors are used to cut the plastic bottles and tubings. It was found that score of ‘mutilation of recyclable waste’ in Treatment Room of wards was significantly lower as compared to OTs and Central Waste Collection Points of wards. The Treatment Rooms are used only by resident doctors to perform minor procedures on admitted patients. Poor score of these areas means that they may be more concerned with direct patient care activities and may be less sensitive towards their duty of BMW management posing an avoidable risk to themselves, patients, rag-pickers, general community and environment.

Further analysis of scores of individual parameters that constituted the category ‘mutilation of recyclable waste’ showed that HCWs do not bend used needles manually; they very rarely re-cap the used needles and generally mutilate the used hypodermic needles. However, they lay less emphasis on mutilation of nozzle of used syringes. They pay even less attention to cutting of used plastic bottles and tubings.

World Health Organization (WHO) has stated that “In unregulated environment, elaborate enterprises have grown up to divert used syringes from waste stream for reprocessing and sale back into unsuspecting markets” [15]. It makes it essential to mutilate used recyclables right after use thus leaving no scope for their unauthorized re-circulation and inappropriate reuse. Providing training is considered an effective tool to increase compliance to guidelines for waste management [16] and so HCWs especially resident doctors may need to be provided training in systematic manner so that they may pay more attention to proper management of BMW.

Most of other studies have also shown poor management of sharps. In a study in Pakistan, practices of poor disposal of sharps were found as 60% of observed practitioners were found throwing syringes at open places [17]. Practices of poor disposal of sharps were also found in a province of China as 8.9 to 23.3% of HCWs were disposing off used needles and syringes in an inappropriate manner [18]. In a hospital at Indore, Madhya Pradesh, India, there were found to be good practices of mutilation of used hypodermic needles and syringes [19]. However, authors have not mentioned to what extent, the practices were followed.

Relatively lower score of category ‘disinfection of waste’ as compared to other categories of waste management indicates that this aspect of waste management is generally overlooked by HCWs. Amongst the various areas, the significantly lower score in treatment room of wards indicates that the resident doctors who use treatment room pay little attention to the disinfection of waste. This may be due to the same reason as mentioned above for ‘mutilation of recyclable waste’ i.e. they might be considering providing direct medical care to patients as their primary duty not appreciating the indirect health risks that they pose, particularly to the waste-handlers, by not disinfecting waste and so need for their training for proper waste management is again highlighted.

Various studies have mentioned that HCWs were using chlorine solution [6,13] or autoclave [19] to disinfect the waste, however, the authors have not mentioned the extent of compliance by HCWs. In the hospital under study, the standard practice was to use chlorine solution in the form of 1% bleaching powder solution for disinfection purposes. The finding of lower score for this category of waste management means that special focus may need to be laid on this aspect while imparting training to HCWs.

## Conclusions

The present study was done to evaluate the practices of biomedical waste management amongst different patient care areas in tertiary care medical institute of North India using a checklist. It was found that more emphasis needs to be laid for ‘mutilation of recyclable waste’ and disinfection of waste’ especially in ‘Treatment Room of wards’ which are used exclusively by resident doctors. Hospital administrators may need to formulate and implement a plan for providing appropriate training to HCWs especially resident doctors so as to address the deficiencies observed in the study.

## Competing interests

The authors declare that they have no competing interests.

## Authors’ contributions

RK designed the study, collected data and drafted the manuscript. AKG participated in design, drafting and coordination; AKA conceived the study, performed data analysis and helped in drafting the manuscript. AK participated in design and helped in drafting manuscript. All authors read and approved the final manuscript.
